# Parasites and vectors carry no passport: how to fund cross-border and regional efforts to achieve malaria elimination

**DOI:** 10.1186/1475-2875-11-344

**Published:** 2012-10-11

**Authors:** Cara Smith Gueye, Alexandra Teng, Kelvin Kinyua, Frank Wafula, Roly Gosling, David McCoy

**Affiliations:** 1 Global Health Group, University of California, San Francisco, USA; 2 San Francisco School of Medicine, University of California, San Francisco, USA; 3 Aidspan, Nairobi, Kenya; 4 Centre for Primary Care and Public Health, Queen Mary University, London, UK

**Keywords:** Malaria, Elimination, Global Fund, Financing, Funding, Proposals, Multi-country, Cross-border, Regional

## Abstract

**Background:**

Tremendous progress has been made in the last ten years in reducing morbidity and mortality caused by malaria, in part because of increases in global funding for malaria control and elimination. Today, many countries are striving for malaria elimination. However, a major challenge is the neglect of cross-border and regional initiatives in malaria control and elimination. This paper seeks to better understand Global Fund support for multi-country initiatives.

**Methods:**

Documents and proposals were extracted and reviewed from two main sources, the Global Fund website and Aidspan.org. Documents and reports from the Global Fund Technical Review Panel, Board, and Secretariat documents such as guidelines and proposal templates were reviewed to establish the type of policies enacted and guidance provided from the Global Fund on multi-country initiatives and applications. From reviewing this information, the researchers created 29 variables according to eight dimensions to use in a review of Round 10 applications. All Round 10 multi-country applications (for HIV, malaria and tuberculosis) and all malaria multi-country applications (6) from Rounds 1 – 10 were extracted from the Global Fund website. A blind review was conducted of Round 10 applications using the 29 variables as a framework, followed by a review of four of the six successful malaria multi-country grant applications from Rounds 1 – 10.

**Findings:**

During Rounds 3 – 10 of the Global Fund, only 5.8% of grants submitted were for multi-country initiatives. Out of 83 multi-country proposals submitted, 25.3% were approved by the Technical Review Panel (TRP) for funding, compared to 44.9% of single-country applications. The majority of approved multi-country applications were for HIV (76.2%), followed by malaria (19.0%), then tuberculosis (4.8%). TRP recommendations resulted in improvements to application forms, although guidance was generally vague. The in-depth review of Round 10 multi-country proposals showed that applicants described their projects in one of two ways: a regional ‘network approach’ by which benefits are derived from economies of scale or from enhanced opportunities for mutual support and learning or the development of common policies and approaches; or a ‘cross-border’ approach for enabling activities to be more effectively delivered towards border-crossing populations or vectors. In Round 10, only those with a ‘network approach’ were recommended for funding. The Global Fund has only ever approved six malaria multi-country applications. Four approved applications stated strong arguments for a multi-country initiative, combining both ‘cross-border’ and ‘network’ approaches.

**Conclusion:**

With the cancellation of Round 11 and the proposal that the Global Fund adopt a more targeted and strategic approach to funding, the time is opportune for the Global Fund to develop a clear consensus about the key factors and criteria for funding malaria specific multi-country initiatives. This study found that currently there was a lack of guidance on the key features that a successful multi-country proposal needs to be approved and that applications directed towards the ‘network’ approach were most successful in Round 10. This type of multi-country proposal may favour other diseases such as HIV, whereas the need for malaria control and elimination is different, focusing on cross-border coordination and delivery of interventions to specific groups. The Global Fund should seek to address these issues and give better guidance to countries and regions and investigate disease-specific calls for multi-country and regional applications.

## Background

Malaria has been and remains a global challenge. Tremendous progress has been made in the last ten years in reducing morbidity and mortality caused by malaria [1]. From 2000 to 2009, there has been an estimated 17% global reduction of malaria incidence, from 225 million cases of malaria in 2000 [1] to 216 million in 2010 [2]. Deaths attributed to malaria have declined by 32% between 2004 and 2010 [3]. Today, many countries are striving for malaria elimination - the interruption of transmission of all *Plasmodium* parasites within their borders [4]. This substantial progress has been made possible because of major improvements in global funding for malaria control and elimination over the last decade. The region of sub-Saharan Africa is a good example of this trend. In 34 countries of this region, there was a 66-fold increase in the amount of official development assistance (ODA) disbursed for malaria control, from $9.8 million in 2002 to $651.7 million in 2008 [5]. This increased funding has led to the scale-up of improved treatment through artemisinin-combination malaria therapy (ACT), improvements in diagnosis and increased coverage of vector control tools such as long-lasting insecticide treated nets (LLIN) and indoor residual spraying (IRS).

Amidst this progress, however, a number of challenges remain, including the relative neglect of cross-border and regional initiatives in malaria control and elimination. Border regions are often overlooked and hinder the goal of malaria elimination. Frequent human and vector border movements, a blurring of responsibility of individual countries in these regions, and relatively poorer access to health care and preventative measures, in particular for mobile populations, leave space for reservoirs of infection that can lead to continued low level transmission of malaria and vulnerability to malaria outbreaks and epidemics.

Such problems are experienced in a number of continents. In southern Africa, movements across the Angola-Namibia, Zimbabwe-South Africa and Mozambique-Swaziland borders create challenges for successful malaria elimination. In Swaziland, 90% of imported cases of malaria across two years (2005–2007) came from Mozambique [6], from groups such as truckers, informal traders and sugar plantation workers [7]. In the northern South African province of Mpumalanga, from 2001 to 2009, 48% of the cases were acquired from Mozambique [8]. In Latin America, cross-border movement of people between Argentina, Paraguay and Brazil is essentially preventing both Argentina and Paraguay from declaring themselves free from malaria. It is estimated that 50 to 70% of all reported cases in Argentina are linked to migration, in particular movement across the border from Bolivia [9,10]; this migration is fueled by economic growth on the Argentina side and a porous border between countries [11]. Similarly, cross-border population movement in the Greater Mekong Subregion of South East Asia impedes efforts to prevent the spread of drug-resistant malaria. An example of this challenge is in Yunnan Province of China, where in 2009 98.8% of total malaria cases and 75.0% of *P. falciparum* malaria cases were found to be imported from neighbouring countries [12]. Across the border in Myanmar there is poor access to malaria control interventions which is contributing to the continuing transmission in this area.

Given these persistent challenges along and across borders around the globe, one solution is to create a multi-country regional initiative. These initiatives can accelerate progress towards malaria elimination by increasing the scale and quality of malaria control activities. There are many examples where countries direct large amounts of internal and external resources toward combatting imported malaria: Suriname targets gold mining groups from Brazil, public hospitals in Bhutan treat people from Assam, and China implements control measures along the Yunnan Province-Myanmar border in order to prevent, diagnose and treat malaria in migrants [13-15]. All of these activities are currently or have been supported by the Global Fund in addition to national resources. These expenditures potentially could have higher impact if conducted as multi-country initiatives in that the funding and supervision could take place in all affected countries. Surveillance systems and methods of inter-country communication could be built to benefit both countries and vector control methods, insecticides, and timing of activities could be harmonized, increasing the potential for reduction in transmission and reducing the potential of insecticide resistance. Research could inform better identification of migration trends and targeting of the populations. Through these activities, regional collaborations can increase the quality and scale of interventions in a highly-endemic neighbour while at the same time reducing risk of importation and potentially the cost of malaria control in the low-endemic country.

In this way, multi-country initiatives can add value to prevention and control of infectious disease, in particular across porous borders. This value can be seen in various current experiences. Due to the combined effort of the Lubumbo Spatial Development Initiative (LSDI) of Mozambique, South Africa, and Swaziland, funded by the Global Fund to Fight AIDS, Tuberculosis and Malaria (Global Fund) malaria prevalence in targeted areas has been reduced by more than 90% [6,16]. The Amazonian Malaria Initiative (AMI), funded by USAID, consists of national ministries of health and technical partners of seven countries is similarly on the path towards successful malaria eradication [17]. In the Middle East, Saudi Arabia, Kuwait, Oman, United Arab Emirates, Bahrain, and Qatar have combined efforts to donate $17 million (USD) towards malaria elimination interventions along the Yemen border to support regional efforts for malaria control and elimination [18].

While regional and cross-border programmes are recognized as being important for the control and eventual elimination of malaria, they do not always receive the required political or financial support. This may be because border areas are susceptible to political instability or conflict; and because it can be difficult to broker fair, acceptable, and sustainable agreements amongst different countries to co-fund or co-manage cross-border programmes. For example, despite it success thus far, LSDI currently does not have long-term funding support beyond 2012. Likewise, the Yemen-Saudi cross-border malaria control programme is threatened by political instability.

For these reasons, the role of the Global Fund in supporting regional or cross-border malaria control efforts is important. The Global Fund is an international financing agency established to support HIV, tuberculosis and malaria programmes in mainly low and middle-income countries. For the past ten years, countries have submitted funding proposals to the Global Fund during periodic ‘rounds’ of funding which occurred approximately once every year. These proposals are reviewed by a Technical Review Panel (TRP) consisting of up to 42 experts that makes a recommendation to the Global Fund Board about whether the proposal should be funded. The Global Fund also allows countries to develop and submit multi-country proposals so as to enable economies of scale and learning across countries, as well as cross-border work. Multi-country proposals can be of two types: a group of small island nations too small to make individual applications or a group of contiguous countries who come together for a regional or cross-border collective effort.

While the Global Fund is not the only source of funding available for these multi-country projects, its contributions to overall malaria programme financing in low- and middle-income countries has been estimated to be 45%, well above that of other donors, whether multi or unilateral [2]. Thus the GF plays a significant role in determining which multi-country projects get funded overall. For this reason, this review concentrates on the Global Fund’s role in financing multi-country initiatives. Given the importance of regional and cross-border initiatives in the future of malaria control, this review aimed to achieve a better understanding of the Global Fund’s support for such initiatives in terms of: 1) the Global Fund’s policies and practices for funding multi-country proposals; 2) temporal trends in the submission and funding of multi-country proposals to the Global Fund; and 3) the factors and variables associated with successful proposals.

## Methods

In order to describe and understand the Global Fund’s policies and practices for funding multi-country proposals, relevant literature was collated from the Global Fund website [19] and Aidspan [20] (a non-governmental, Kenya-based organization whose mission is to reinforce the effectiveness of the Global Fund by monitoring it and providing information about the Global Fund to potential applicants). A number of Global Fund and Aidspan documents were reviewed (see Table 1).

**Table 1:** List of documents reviewed

**Type of document**	**Rounds available**	**Type of information**
***Global Fund documents***		
Technical Review Panel reports	2-10	Released after review of proposals by the TRP; summary of approved and not approved proposals; recommendations to Board and Secretariat
Frequently Asked Questions	6-9	Released by GF during call for proposals; guidance on developing and submitting a proposal
Fact Sheets	8, 9	Released by GF during a call for proposals; each focuses on a certain approach (e.g., health systems strengthening)
Information Notes: Multi-Country Applicants	9, 10	Released by GF during call for proposals; focuses on specific guidance for multi-country applications
Guidelines for Proposals: Multi- Country Applicant	10	Released by GF during the call proposals; guiding principles to Global Fund applicants with information on each question in the proposal
Proposal template – for all applicants	2-7	Released during the Call for Proposals
Proposal template – for multi-country applicants only	8-10	Released during the Call for Proposals
***Aidspan documents***		
Guide for Multi-Country Applications	10	

Data on temporal trends in grant approval rates by proposal type (single and multi-country) and by disease were obtained from the Global Fund website grant data [21], Global Fund approved proposals available on the website [22], and Aidspan *Global Fund Observer* documents [23].

In order to describe and understand the factors associated with successful multi-country applications, we first examined all Round 10 multi-country applications for three diseases (HIV, malaria and tuberculosis), of which there were 15. Then we reviewed successful malaria multi-country grant applications from Rounds 1 to 10, of which there were six. Only four were reviewed as described in the findings (below).

Each application was reviewed and examined against a list of 29 variables organized according to eight dimensions (see Additional file 1). Eight general dimensions were developed from several sources: background papers on malaria elimination, cross-border challenges, and the Global Fund Round 10 multi-country proposal template. Within those eight dimensions, the 29 variables were developed based on factors that characterize a Global Fund grant proposal, including the content, technical quality, regional context, epidemiological features, and the nature of the applicant and Principal Recipient. Thirteen variables (1–4, 7–10, 14, 15, 23, 24, and 29) were considered objective in that they had yes/no or multiple choice answer fields, while sixteen variables (5, 6, 11–13, 16–22, 25–28) were considered subjective in that they required interpretation by the reviewer to justify a categorical rating. Based on what background research described as the most important reasons for cross-border approaches, it was predicted that variables 19, 20 and 21 would be the key factors determining the success of an application.

One reviewer (AET), blinded to the Global Fund decision of each proposal, conducted a review of all 15 multi-country Round 10 applications. The other members of the research team (CSG, DM, and RG) reviewed two randomly selected applications. The research team then discussed their findings and differences in the way they applied the framework until a shared view was developed of how to interpret funding proposals and apply the framework of variables to them.

The primary reviewer (AET) then reviewed the remaining Round 10 applications after which the categorical variables were scored and tallied and differences between the approved and not approved Round 10 applications were noted. The comments provided for each qualitative indicator were also examined for any differences or trends between approved and not approved applications. AET then reviewed the successful malaria multi-country proposals from Rounds 1–9, as a review of all Round 10 applications was already conducted during the first phase of this research.

## Findings

### Global Fund policy and guidance

Starting with Round 1, Global Fund accepted proposals from both single and multi-country applicants. Global Fund policy, guidance and procedures for multi-country applications have naturally evolved over time. After each round of applications, the TRP would issue a report to the Global Fund making recommendations for how the process could improve, and this included specific recommendations about multi-country applications. For example, after Round 2, a TRP recommendation for all multi-country applications to be explicitly endorsed by the Country Coordinating Mechanism (CCM) for all countries in a multi-country proposal [24] was implemented for Round 3 and subsequent rounds [25]. The CCM is a country-level multi-stakeholder partnership that develops and submits grant proposals to the Global Fund, then oversees progress during implementation [19].

In the very early rounds, multi-country applicants were required to use the same forms as single country applicants. In Rounds 2 and 3, there was only one question posed specifically to multi-country applicants, which was to describe the added value of a multi-country application. In Round 4, multi-country applicants were required to answer additional questions about consistency with national plans and strategies (this followed a recommendation from the TRP).

From Rounds 4–6, the Global Fund attempted to address some of the problems identified by the TRP. These included the lack of evidence provided about the added value of a multi-country initiative (R4), the expensive budgets being allocated for administrative purposes (R5,6), and the “opportunistic” creation of multi-country initiatives that may “serve the needs of the implementing organizations rather than the countries” [26]. In R6 the TRP also noted that many multi-country applications were technically unsound and posed a risk of undermining existing programmes and national health systems [27]. Following Round 6, the TRP suggested a major review and revision of the proposal form and guidelines.

As a consequence, the applications forms for Round 7 contained an entire section that was dedicated to multi-country applications, which required more comprehensive information about the need for a multi-country or regional application, the added value of the application and the mechanism for the coordination of activities across the different countries [28].

For Rounds 8–10, the Global Fund introduced a separate and specific application form for multi-country applicants. However, some sections on the new form were less specific than previous versions. For example, the questions that attempted to draw out a clear description of the rationale for a multi-country rationale were less rigorous in Rounds 8–10 than in Round 7. In Round 7 the proposal challenged the applicant to describe “why a multi-country proposal will be more effective in responding to the issues presented than if each CCM presented the same activities on a country by country basis…” Whereas in Rounds 8, 9, and 10, the question is broader, only seeking to have the “rationale for a multi-country approach” through a “brief overview” for the issues described in the proposal.

In Round 8, the TRP also found that multi-country applications that were based on a single Regional Organization delivering the activities were weaker than those based on a regional coordination mechanism of multiple organisations. Countries appeared to be included in the Regional Organization proposals because of their eligibility per the Global Fund requirements rather than regional-based needs or epidemiological contexts. The TRP, therefore, recommended for the Board to consider revising eligibility requirements for multi-country proposals, and to specifically review the eligibility criteria for Regional Organizations. However, this did not lead to any changes of the application form or other materials for Round 9.

Following Round 9, the TRP questioned the appropriateness of service delivery in regional proposals and the risk this posed to creating parallel structures of health delivery. Also in Round 9, the TRP noted that countries were not alerting the CCM of their participation in concurrent applications, either in single or multi-country applications [29]. In response to the second challenge, in Round 10 there was an additional table outlining the Service Delivery Areas (SDAs) and how previous and current proposals contribute.

Guidance to multi-country applicants for Rounds 6–9 was provided in the form of FAQ documents. However, these focused on eligibility requirements, such as the proportion of countries qualifying as Upper Middle Income included in the proposal, or the type of applicant that can submit a multi-country proposal [30-33]. More detailed Information Notes for Multi-Country Applicants were made available in Rounds 10 and 11 and these provided guidance on the priority sections of the application form and described the strengths and weaknesses of previous applications [34,35]. A new section in the 2011 Information Note was entitled “type of activities considered inappropriate for Regional proposals” and provided for the first time an explicit description of the kinds of activities that were deemed only appropriate to fund through a single-country application. One such activity was “delivering services directly in country.”

The Global Fund issued guidelines for multi-country applicants starting in Round 10. These guidelines gave question-by-question advice. While this guidance appears to be very useful for the summary and rationale for multi-country approach, many sections duplicated the same information for single-country applicants (e.g., improving value for money) [36].

### Trends in multi-country applications Rounds 3–10

During Rounds 3–10 of the Global Fund, there were an estimated 1,419 grant applications received by the Global Fund. Only 83 of these applications were for multi-country applications (5.8%), the rest being single-country applications. Rounds 1 and 2 are not included in these figures because the numbers of submitted applications were not available by disease category. There was no obvious temporal trend noted in the overall number of multi-country applications submitted, although Rounds 5, 7, and 8 had the lowest number (Table 2). There was no clear trend in applications by disease either. Of the 83 total multi-country applications submitted from Rounds 3 to 10, only 21 were approved by the TRP for funding (25.3%) compared to 600 (44.9%) single-country applications.

**Table 2:** Multi-country proposals submitted and results

**Multi-country proposals submitted and results**						
**Round**	**HIV submitted**	**HIV approved**	**Malaria submitted**	**Malaria approved**	**TB submitted**	**TB approved**
1	NA	3	NA	0	NA	0
2	NA	1	NA	2	NA	1
3	8	2	6	1	1	0
4	8	2	3	0	0	0
5	4	0	2	3*	1	0
6	8	1	2	0	0	0
7	7	2	0	0	1	1
8	4	0	1	0	0	0
9	11	4	1	0	0	0
10	12	5	1	0	2	0

Note: For Rounds 1–2, information is available only on approved proposals.

*One (single) proposal resulted in two grants in Round 5.

From Rounds 3 to 10, the majority of multi-country applications submitted were for HIV (74.7%), followed by malaria (19.3%) and then by tuberculosis (6.0%). The majority of approved multi-country applications were for HIV (76.2%) followed by malaria (19.0%), then tuberculosis (4.8%). The approval rate for multi-country applications across Rounds 3 to 10, by disease category, was: 25.8% for HIV; 25.0% for malaria; and 20.0% for tuberculosis.

### In depth review of round 10 multi-country proposals

The Global Fund received a total of 15 multi-country applications for Round 10, of which five were recommended for funding. There was only one multi-country application submitted for malaria, which was not approved. All five of the successful multi-country applications were for HIV/AIDS programmes (see Table 2). Of the 15 applications submitted, one was submitted as a Type A proposal, or a group of small island nations and was not approved. The other 14 were submitted as a group of contiguous countries, or as a Type B proposal.

There were general attributes noted across all applications. First, all 15 multi-country proposals had a community systems strengthening (CSS) component. Second, in all the applications, the added value of a multi-country initiative was described in terms of one of two arguments: 1) a regional ‘network approach’ by which benefits are derived from economies of scale or from enhanced opportunities for mutual support and learning or the development of common policies and approaches; or 2) a ‘cross-border’ approach for enabling activities to be more effectively delivered towards border-crossing populations or vectors.

There were nine applications that demonstrate the network approach, of which 5 were funded. In one application, a non-governmental organization (NGO) regional network across four countries sought to strengthen advocacy actions of community-based organizations to achieve improved access to comprehensive HIV interventions [37]. The application described efficiency gains and cost savings as a result of sharing strategies and training across organizations. In another application, an international network of people living with HIV submitted a proposal to administer sub-grants to national networks of people living with HIV that would enable them to provide services, fundraise, and improve programme management skills [38]. A third application, which was not approved, similarly was aiming to work with national and sub-regional transgender organizations and their leaders, except as direct recipients, to improve social climate, strengthen capacities and build evaluation and monitoring systems [39]. This project aimed to ultimately benefit the region’s population.

There were six multi-country grant applications that were focused on the cross-border approach, none of which were approved. One application focused on HIV prevention by targeting long-distance truck drivers and at-risk populations at roadside truck stops close to national borders [40]. Another application focused on remote border regions between three countries and the large-scale movement of poverty-stricken people through the area (generally from high to low malaria transmission areas) [41]. The populations were described as lacking access to prevention, diagnosis and treatment and as being subject to discrimination.

Other trends were identified. Applications that included prevention and health services delivery as major activities were less likely to be approved than applications which emphasized other activities such as strengthening surveillance systems or strengthening networks. This finding echoes the Round 9 TRP report in which it questioned the appropriateness of funding service delivery through a multi-country grant [35]. Unsuccessful applications also tended to provide limited information on how the proposal was developed and what lessons had been learned when compared with successful applications. The five approved proposals included a detailed description of lessons learned and applied through a Regional Organization or a Principal Recipient that appeared to have significant experience in this type of initiative.

### In depth review of Malaria Multi-country proposals

From Rounds 1 to 10, the Global Fund has only ever approved six malaria multi-country applications. Three of these applications were submitted as a type of multi-country proposal of a group of small island nations (Type A). The remaining three were submitted as a group of contiguous countries (Type B). These applications were submitted in Rounds 2, 3, and 5 as follows:

• Round 2: “Malaria control in the Lubombo Spatial Development Initiative Area (LSDI)” in the border region of Mozambique, South Africa and Swaziland - Type B proposal;

• Round 2: “Pacific Islands Regional Coordinated Country Project on HIV/AIDS, TB and Malaria” for 11 countries (Cook Islands, Federated States of Micronesia, Fiji, Kiribati, Niue, Palau, Samoa, Solomon Islands, Tonga, Tuvalu, and Vanuatu) - Type A proposal;

• Round 3: “Malaria control in the cross-border areas of the Andean Region: A community-based approach” comprised of countries Columbia, Ecuador, Peru, and Venezuela - Type B proposal;

• Round 5: “Multi-Country Response to Malaria in the Pacific” from Solomon Islands and Vanuatu - Type A proposal. There were two proposals submitted in this application;

• Round 5: “Regional proposal for extension of malaria control to Gaza Province, Mozambique as part of the Lubombo Spatial Development Initiative” from Mozambique, South Africa, and Swaziland - Type B proposal.

The second application listed above, which was a Type A proposal on behalf of a group of small islands, was not reviewed as part of the malaria multi-country approved proposals because it was deemed too difficult to tease apart the strengths of the malaria component from the other disease components. One of the two Round 5 “Malaria in the Pacific” proposals was reviewed because the proposals are identical (although the proposal has two grant record numbers, one for each country).

Thus, the four successful malaria multi-country applications were examined using the framework described earlier. Each application was found to have crafted a strong argument for a multi-country initiative, combining both a rationale for a ‘cross-border approach’ (involving mobile populations) and a ‘network approach.’

The application covering the Amazon region, for example, described how the movement of local indigenous groups and vectors bore no relation at all to the construction of national boundaries. The applications also provided strong descriptions of past experience with successful applications or projects. Three of the applications included elements of service delivery, such as scale-up of IRS activities or training of community health workers.

## Discussion

Given the more austere current climate for the funding of malaria, as well as HIV and tuberculosis, it is a timely moment to discuss the purpose, benefit and funding of multi-country and cross-border initiatives for malaria control. The end game with respect to malaria elimination will rely on cross-border and regional initiatives. The many examples of malaria migration across Latin America, Southeast Asia and Southern Africa speak to the challenges faced by low endemic countries in controlling and managing infections across national borders. It is therefore important to understand how the Global Fund approaches the funding of multi-country proposals for malaria treatment and control, and why there is a relatively low success rate for multi-country proposals. This study suggests that there may also be a lack of clarity and guidance about when a multi-country and cross-border approach to disease control is indicated or how the Global Fund should be used to support such approaches. The Global Fund is of course not solely responsible for generating multi-country proposals and programmes – this work is a responsibility of various actors that include the Global Fund but also consists of countries, the World Health Organization, and key malaria-specific donors. However, the Global Fund can play an important role by ensuring that the guidelines and procedures for multi-country proposals are optimal and geared towards funding programmes of high impact and strategic value.

This review found an apparent lack of active encouragement of multi-country applications from those regions of the world where cross-border activities are important for malaria control and elimination. This holds true for both types of multi-country applications, those from a group of small islands or those from a group of contiguous countries for a regional or cross-border effort. In this study we found that applications that were recommended for funding were more likely to be for a “network approach.” While such an approach may be useful and valid for HIV, a cross-border approach is more relevant for malaria control and elimination programmes.

Unlike HIV multi-country proposals, where networking is relevant, malaria control and elimination requires targeting vulnerable, marginalized border-crossing groups and the vulnerable zones through which they move. In order to reach these populations for prevention, diagnosis and treatment of malaria, a funding mechanism is needed to support direct co-ordination of national malaria control programmes, the communities themselves and involve buying commodities and delivery of interventions.

The review of the Round 10 multi-country applications suggests a possible bias in favour of ‘network’ approaches usually based on a rationale of improved efficiency and economies of scale, as well as the argument that marginalized groups would be better empowered through the establishment of trans-national coordination and solidarities. None of the successful Round 10 applications were based upon tangible ‘cross-border’ issues.

In this study of the Global Fund’s funding of multi-country malaria programmes, a relatively small number of multi-country malaria grants that were approved were identified: only six in total over a ten year period. Multi-country malaria submissions were few: only 16 malaria proposals over seven years (Rounds 3–10), compared to 62 over the same period for HIV. There was a low average success rate for all multi-country applications (25.3%) compared to single country successful proposals (44.9%) from Rounds 3–10, of which malaria proposals achieved a 25.0% approval rate similar to 25.8% for HIV and 20.0% for tuberculosis. Given the low success rate of all multi-country proposals as compared to single-country applications, there may be a gap between the information and guidelines put forth by the Global Fund and the comprehension by applicant countries and initiatives.

While there were improvements over time with guidance to support multi-country applications, the Global Fund could do more to facilitate these changes. TRP recommendations resulted in some improvements to the application form, including the development of a new separate form for multi-country applications introduced in Round 8. But the Global Fund Information Notes were generally vague in guidance, even though the 2011 notes were much improved. There is still a gap in information from the funder for applicants as to how the multi-country applications can support context-based regional needs for cross-border public health interventions or prevention activities, such as vector control in an area of vulnerability. Applicants need more comprehensive guidance on the type of projects, activities or situations that are best suited for multi-country proposals. Success stories or examples of functioning and productive multi-country initiatives could help to fill this gap.

### Limitations

This study had several limitations. While the review explored and actively sought to minimize inter-rater variability in the assessment of proposals and the application of the framework, there remains a potential for proficiency bias because the main reviewer (AET) may have differing views and experience level from the other members of the research team, and the entire research team’s expertise may differ from the Global Fund members who evaluate applications. Nonetheless, in spite of the potential limitations of the methodology employed, the exercise has produced some relevant and useful findings. However, should the Global Fund or any other agency seek to use the methods and framework for any future evaluation of multi-country proposals, it would be advisable to conduct a more structured validation process.

In addition, complete documents for unsuccessful multi-country malaria grant applications from Rounds 1–6 or approved applications from Rounds 1 and 2 were not accessible. Representation of multi-country malaria grant applications in the in-depth review of Round 10 was limited to only one application, unlike previous rounds which had more applications. The study also did not interview any members of the Global Fund Secretariat or TRP to gain a better understanding of the factors considered important during assessment of grant applications. Future analysis could include in-depth reviews of TRP members in order to identify important factors that influence decisions for Global Fund applications. Potential applicants for Global Fund funding could be interviewed to understand their perception of multi-country initiatives.

## Conclusions

With the replacement of Round 11 with a Transitional Funding Mechanism and the establishment of a new funding model for 2012 to 2016, the time is opportune now for the Global Fund to develop clearer guidelines for funding malaria-specific multi-country initiatives. Technical agencies could work with the Global Fund to identify those regions of the world where cross-border approaches to malaria control are essential for malaria control and actively facilitate the development of appropriate grant applications for the future. Certain cross-border contexts could greatly benefit from external Global Fund support, including regions with a high disease burden, conflict and political instability, or those contending with the challenge of artemisinin resistance. In some of these areas, active support from United Nations technical agencies might help to facilitate the development of a neutral and appropriate public health infrastructure for the delivery of key health services. This approach could be replicated for HIV and tuberculosis as well, where appropriate.

## Competing interests

CSG is a Program Coordinator and RG is Lead of the Malaria Elimination Initiative at the UCSF Global Health Group, which is funded by the Bill & Melinda Gates Foundation. DM, KK, and FW are supported by Aidspan.org. AET received funding for this project from the UCSF Dean’s Summer Research Fellowship.

## Authors contributions

All authors contributed by guiding and shaping the messages and ideas contained in this paper. The text was drafted by CSG, RG, DM, and AET. AET, CSG, and DM conducted the review and analysis, with assistance from KK and FW. All authors took part in the review, preparation, and final approval of the manuscript.

## Supplementary Material

Additional file 1 **Variables used for Global Fund multi-country proposal review. **This list is comprised of 29 variables of eight dimensions. Each multi-country proposal in the review was reviewed against the variables in this list. Click here for file
