# Supplementary material for: Parasites and vectors carry no passport: how to fund cross-border and regional efforts to achieve malaria elimination
Source: Malar J. 2012 Oct 11;11:344. doi: 10.1186/1475-2875-11-344 (PMC3506506; doi:10.1186/1475-2875-11-344)
Supplement: Additional file 1 — Variables used for Global Fund multi-country proposal review. This list is comprised of 29 variables of eight dimensions. Each multi-country proposal in the review was reviewed against the variables in this list. [file 1475-2875-11-344-S1.docx]

Additional file 1

| Geography and socio-economic characteristics   1. Region [East Asia & Pacific/Eastern Europe & Central Asia/Latin America & Caribbean/North Africa & Middle East/South America/Southeast Asia/South Asia/SSA: East Africa/SSA: Southern Africa/SSA: West & Central Africa/East, Central and Southern Africa] 2. Income classification [Listed each country in the proposal as Low/Low-middle/High-middle/High]   Grant and donor history   1. Is this proposal a resubmission? [Yes/No/No info] 2. Existing or previous Global Fund Grants in country and disease category [Yes/No] 3. Alignment with previous grants (single and/or multi-country and considers lessons learned [Yes-Strong/Yes-Weak/No info] 4. Coordination with the current/planned work of the respective national CCMs or structures of other donor funding [Yes-Strong/Yes-Weak/No info] 5. Evidence of co-funding [% of each country] 6. Proposal term funding amount [<$1M/1M-5M/5M-10M/10M-15M/>15M]   Applicant   1. Type [Regional Coordinating Mechanism/Regional Organization} 2. If Regional Coordinating Mechanism, what sector best describes this organization? [Academic or educational/Government/NGO or CBO/People living with the disease/People representing key populations/Private sector/FBO/Multilateral and bilateral development partners in country/Intergovernmental/Other] 3. If Regional Organization, experience in region [Number years working in region/NA] 4. If Regional Organization, experience with target population [Number years working w/ target population] 5. If Regional Organization, experience with disease [Number years working w/ disease] 6. Evidence of CCM endorsement of proposal? [Yes – all countries represented/Yes – partial country representation/No country representation/No info]   Principal Recipient   1. Type [CS-PS:FBO/CS-PS:NGO/CS-PS:OTHER/CS PS:PS/GOV:MOH/GOV:MOF/GOV:OTHER/MO:UNDP/MO:OTHER] 2. Experience in region [Number years working in region/NA] 3. Experience with target population [Number years working w/ target population] 4. Experience with disease [Number years working w/ disease]   Cross border/regional/multi-country rationale   1. Rationale for selection of countries for the proposal [Strong/Weak/No info] 2. Description of cross-border issues [Yes-Strong/Yes-Weak/No info] 3. Demonstration of the added value of a cross border/regional application beyond that which could be achieved on an individual country basis? [Yes-Strong/Yes-Weak/No info] 4. Potential to duplicate/distort/create parallel structures within each country’s health system delivery [Yes-Strong Potential with comment field/Yes-Some Potential with comment field/No Potential with comment field/No info]   Epidemiology   1. Disease Component [HIV/HIV MARPS/TB/MALARIA] 2. If countries in the proposal are considered Upper Middle Income, is disease category qualified as high-burden? [Yes/No] 3. Does the proposal focus on population groups which are excluded from national strategies or activities (e.g. stigmatized and vulnerable populations or mobile / transient populations) [Yes/No/No info]   Management arrangements   1. Clear description of the division of roles and responsibilities [Yes/No] 2. Are there tangible mechanisms in place for activity coordination? [Yes-Strong/Yes-Weak/No info] 3. Clear reporting structure [Yes-Strong/Yes-Weak/No info]   Planned activities   1. Do the proposal objectives include the following activities: [Yes/No]   Advocacy  Capacity- and skill-building across organizations  Community Systems Strengthening  Development, implementation, harmonization of policies across countries  Documentation and information-sharing, best practices  Expansion of knowledge and evidence/base  Health service delivery (bio-medical) outside of pilot projects  Health Systems Strengthening  Prevention services  Social mobilization  Strengthen networks  Strengthen surveillance systems |
| --- |
